# In silico validation of electrocardiographic imaging to reconstruct the endocardial and epicardial repolarization pattern using the equivalent dipole layer source model

**DOI:** 10.1007/s11517-020-02203-y

**Published:** 2020-05-31

**Authors:** Jeanne van der Waal, Veronique Meijborg, Steffen Schuler, Ruben Coronel, Thom Oostendorp

**Affiliations:** 1 Department of Clinical and Experimental Cardiology, Amsterdam University Medical Centers, Meibergdreef 9, 1105 AZ Amsterdam, The Netherlands; 2 Institute of Biomedical Engineering, Karlsruhe Institute of Technology, Fritz-Haber-Weg 1, 76131 Karlsruhe, Germany; 3 Donders Institute for Brain, Cognition and Behaviour, Radboud University Medical Centre, Kapittelweg 29, 6525 Nijmegen, The Netherlands

**Keywords:** Equivalent dipole layer, Electrocardiographic imaging (ECGI), Inverse problem of ECG, Repolarization, Cardiac arrhythmias

## Abstract

The solution of the inverse problem of electrocardiology allows the reconstruction of the spatial distribution of the electrical activity of the heart from the body surface electrocardiogram (electrocardiographic imaging, ECGI). ECGI using the equivalent dipole layer (EDL) model has shown to be accurate for cardiac activation times. However, validation of this method to determine repolarization times is lacking. In the present study, we determined the accuracy of the EDL model in reconstructing cardiac *repolarization* times, and assessed the robustness of the method under less ideal conditions (addition of noise and errors in tissue conductivity). A monodomain model was used to determine the transmembrane potentials in three different excitation-repolarization patterns (sinus beat and ventricular ectopic beats) as the gold standard. These were used to calculate the body surface ECGs using a finite element model. The resulting body surface electrograms (ECGs) were used as input for the EDL-based inverse reconstruction of repolarization times. The reconstructed repolarization times correlated well (COR > 0.85) with the gold standard, with almost no decrease in correlation after adding errors in tissue conductivity of the model or noise to the body surface ECG. Therefore, ECGI using the EDL model allows adequate reconstruction of cardiac repolarization times.

**Electronic supplementary material:**

The online version of this article (10.1007/s11517-020-02203-y) contains supplementary material, which is available to authorized users.

**Graphical abstract:**
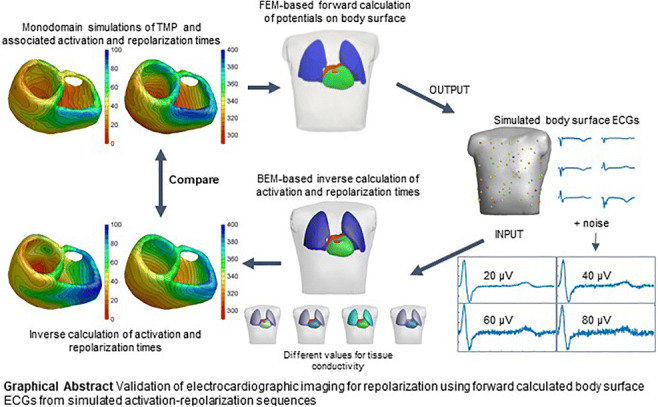
Validation of electrocardiographic imaging for repolarization using forward calculated body surface ECGs from simulated activation-repolarization sequences.

## Introduction

Cardiac ventricular arrhythmias are an important cause of death worldwide [22]. The initiation and maintenance of potentially fatal reentrant arrhythmias is facilitated by regional heterogeneities in activation and repolarization times [5]. Early risk stratification of patients therefore is important to reduce mortality caused by arrhythmias. In addition, non-invasive determination of size and location of heterogeneities can be helpful in deciding on therapeutic strategy.

The solution of the inverse problem of electrocardiology allows the spatial reconstruction of the electrical activity of the heart (electrocardiographic imaging, ECGI) from the body surface ECGs and the patient’s heart-torso geometry. Thereby the ECGI non-invasively determines the sequence of activation and repolarization. Several inverse calculation methods exist. The most often used methods are the epicardial potential model [1, 4, 30, 32] and the equivalent dipole layer (EDL) model [14, 15, 23, 24]. The latter is used in this paper.

The activation wavefront within the myocardium acts as a dipole layer of uniform strength [27]. A uniform dipole layer at the myocardial surface (both epicardium and endocardium) that encompasses the part of the surface that has been reached by activation produces the same potentials at the body surface as the actual activation wavefront (under certain assumptions on the myocardial conductivity) [11]. This is the basis of estimation of activation times using the EDL. Validation studies have shown good accuracy for reconstructing activation times and origins of premature ventricular complexes [15, 24].

The estimation of depolarization sequences is based on an “on-off” scenario, i.e., tissue is either activated or not. Since repolarization occurs gradually, estimation of repolarization sequences cannot be based on this on-off scenario; therefore, the uniform dipole layer approach is no longer applicable. Geselowitz has shown that this theorem can be adjusted to include the complete cardiac cycle by considering a non-uniform dipole layer with a strength that is proportional to the local transmembrane potentials at the surface (provided that the ratio between the conductivity along the fibers and perpendicular to the fibers is the same for the intracellular and extracellular media) [12]. This adjusted theorem to estimate repolarization times has never been adequately tested before.

The inverse problem is *ill-posed* [1, 14], making it sensitive to small perturbations in the measured body surface potentials, such as noise. Since the electrographic T wave has lower amplitudes and less steep slopes than the QRS complex, it may be even more sensitive to noise. Additional errors in accuracy may come from the use of incorrect tissue conductivity values, considering a lack of consensus in the literature about measured conductivity values [8]. These errors influence the forward-calculated ECG [17], and may also influence the inverse calculation of cardiac repolarization times.

We aimed to assess the accuracy of the EDL model in calculating the repolarization times, as well as the method’s robustness by adding noise to the ECGs or adding errors in conductivity of surrounding tissue. The EDL model was applied to simulated electrocardiographic data containing different activation and repolarization patterns produced independently by a detailed propagation and volume conductor model [15, 34].

## Methods

### Anatomical modeling

Thoracic magnetic resonance images (MRI) of a 27-year old healthy volunteer were used as a basis to create an inhomogeneous, anisotropic volume conductor mesh [15]. The torso MRI data had a voxel size of 1 × 1 × 2 mm^3^, while the heart was imaged with the resolution of 1 × 1 × 1 mm^3^. The segmentation was performed in a semi-automatic manner using region growing and active contours methods. The tetrahedral mesh included lungs, thorax, ventricular myocardium, and intracavitary blood volumes of both atria and ventricles. Transmembrane potentials (TMPs) during the complete cardiac cycle were computed using an anisotropic monodomain model [34]. A voxel mesh with a resolution of 0.4 mm (3,019,701 myocardial voxels) was used for this purpose. Nearest neighbor interpolation was then used to transfer the TMPs from the voxel mesh to the tetrahedral heart domain of the volume conductor mesh, consisting of 48,671 nodes (mean edge length: 2.1 mm). This resolution is sufficient to represent TMPs during repolarization, as they are characterized by low spatial frequencies during this phase. These TMPs were used as gold standard for comparison with the reconstructed TMPs. With the obtained TMPs, forward calculation was done using the finite element method (FEM) to compute electrocardiograms at chosen positions on the thorax model. These body surface ECGs were used as input for the EDL-based inverse procedure. The EDL method is a nonlinear optimization routine reconstructing the distribution of activation and repolarization times, given a template for temporal course of the TMP. These inverse calculations were performed independently from the method to compute the ECGs, using the boundary element method (BEM) and surface meshes of the compartments of the geometrical model. In the BEM model-based inverse calculations, the myocardial surface mesh consisted of 1500 ventricular nodes (a subset of the points within the FEM grid) and isotropic bulk conductivity for the myocardium was used (being the sum of the intra- and extracellular conductivities from the FEM model). The repolarization patterns on the ventricular surfaces reconstructed by the EDL inverse method were compared with those extracted from the anisotropic monodomain model simulated TMPs (gold standard) at these 1500 nodes.

**Fig. 1:**
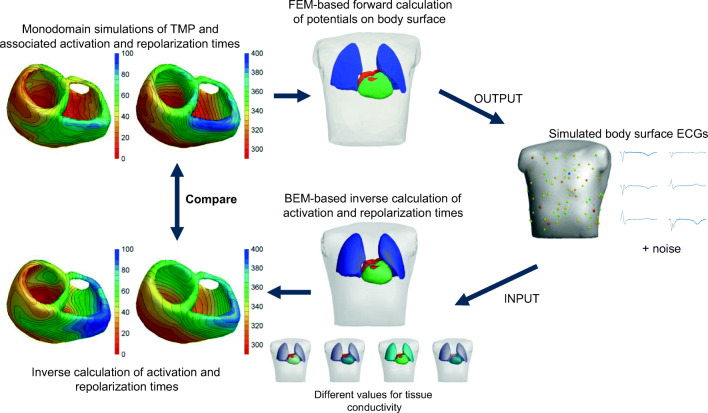
Study workflow scheme. The top part shows how the TMP simulations are used to calculate the body surface ECGs. The bottom part shows how the simulated body surface ECGs are used as an input in the inverse method to reconstruct activation and repolarization times. To test the robustness, we repeated the workflow for different levels of noise added to the input ECGs and for different conductivity sets within the BEM model

The simulation workflow is visualized in Fig. 1, and the various steps of the inverse method are described in more detail below.

### Simulating body surface ECGs

The TMPs were computed by solving an anisotropic monodomain model with the parallel solver acCELLerate [34]. Ionic currents were defined by the ventricular cell model proposed by Ten Tusscher and Panfilov [36]. A rule-based approach was used for creation of the fiber orientation [17], and different heterogeneities for calculation of the TMP distributions were integrated into the model according to Wilhelms et al. [38].

The TMPs were generated for three conditions as follows: sinus rhythm and two ventricular ectopic premature beats, one originating from the interventricular septum and the other from the right ventricular base. For sinus rhythm, a rule-based endocardial stimulation profile imitating the Purkinje fibers was used [16, 17]. For simulating ectopic beats, a spherical area with two voxel radii was stimulated in order to initiate excitation. The computed TMPs throughout the myocardial tissue were subsequently used in the forward FEM calculation of electrocardiograms at 120 electrode positions on the body surface in the FEM thorax model. The volume conductor model included an equal anisotropy ratio for the myocardium, whereby the conductivity values along the myocardial fibers were set to be three times larger than perpendicular ones, and were equal for the intra- and extracellular media [15].

The constructed body surface potential maps were contaminated with 20 μV Gaussian white noise (baseline noise) and served as input for the EDL-based inverse procedure.

### EDL-based inverse

The inverse solution using the EDL source model entails iterative forward calculations of body surface potentials in order to optimize the model parameters (activation and repolarization time on the myocardial surface) by determining the best match with the simulated body surface potentials. Since the reconstructed body surface potentials depend on the model parameters in a non-linear way, the iterative process must apply a non-linear parameter estimation procedure starting from an initial estimate for activation and repolarization time.

#### TMP template

In our implementation of the EDL model, a template for the time course of the TMP was constructed. This template is shifted and stretched to construct the TMP for an individual node to fit its depolarization time τ and the repolarization time ρ. During the QRS complex, the transmembrane potential at the myocardial surface can be considered to be either “at rest” or “activated.” This is implemented in the TMP template by a Heaviside step function (i.e., *H*(*t* − *τ*_*j*_) = 0 for  *t* − *τ*_*j*_ < 0  and *H*(*t* − *τ*_*j*_) = 1 for *t* − *τ*_*j*_ > 0), and is known as phase 0 of the action potential.

The repolarizing part of the TMP template (phases 1, 2, and 3 of the action potential) is individualized by taking the “dominant T-wave”; i.e., the shape of the T-wave from the root-mean-squared body surface potentials. The dominant T-wave reflects the first-order derivative of the averaged transmembrane potential of the myocytes during repolarization, and therefore, its integral describes a generalized transmembrane potential during repolarization [25, 26]. From all body surface potentials resulting from the cardiac activation model, the root-mean-square (RMS) is taken. Based on this RMS signal, the shape of the dominant T-wave is determined. The flipped integral of this dominant T-wave shape provides a template for the repolarizing part of the TMP waveform. This template is rescaled for each individual node at the myocardial surface so that at the repolarization time ρ the TMP has decreased in amplitude by 80%.

#### Computing body surface potentials

The transmembrane potential (based on depolarization time τ and the repolarization time ρ as explained above) is used as source activity to compute the resulting potential *φ*_*i*_(*t*) at electrode position *i* on the body surface at time t:

$$ {\varphi}_i(t)=\sum \limits_{j=1}^{N_{\mathrm{v}}}{A}_{ij}\ {\mathrm{TMP}}_j(t), $$

where *N*_v_ is the number of discretization elements of the ventricular surface, *A*_ij_ indicates the potential generated at *i* by an equivalent dipole layer element of unit strength at element *j* of the ventricular surface, and TMP_j_(t) is the transmembrane potential at location *j* of the ventricular surface determined by activation and repolarization time at *j*. The transfer matrix A was computed using BEM as previously described by Janssen et al. [15].

#### Initial estimate of activation and repolarization

The initial estimate of activation is provided by the fastest route algorithm, as previously described [7, 15]. In brief, each node on the heart surface is considered an initial focus, and corresponding activation times on the heart surface are computed assuming different propagation velocities along the myocardial surface. The activation pattern creating body surface potentials that correlate best with the simulated body surface ECGs is chosen as initial estimate. In the same way, additional foci are added until there is no further significant improvement of the correlation.

The repolarization pattern depends on the depolarization sequence, with generally longer action potential durations in early activated regions and shorter action potential durations in late activated regions [9, 29]. Although this rule of thumb is not always applicable, it is compatible with the common observation in (12-lead) ECGs that the T-wave is concordant with the QRS polarity, indicating that, overall, repolarization occurs in a direction opposite to depolarization direction. Therefore, we used an initial estimate for the repolarization times that is inversely dependent on the depolarization times for sinus beats [6]. However, in a premature ventricular complex, the T-wave is often discordant with the QRS complex, indicating a similar direction of activation and repolarization [33]. Accordingly, we used an initial estimate of repolarization for the ectopic ventricular beats that matches the initial estimate for activation with a time delay equal to the time from the J-point (end of QRS) to the end of the T-wave.

#### Non-linear optimization

Starting from the initial estimates, the depolarization and repolarization patterns were iteratively optimized by minimizing the difference between measured and reconstructed body surface potentials. For this non-linear optimization procedure, we used a dedicated version of the Levenberg–Marquardt algorithm [6, 21]. In each iterative step, optimization of depolarization and repolarization was done alternately. Thereafter, the ECG reconstructed from the inverse estimated activation and repolarization times and the simulated body surface ECG were compared. Iterative optimization continued until no further improvement in ECG correlation was detected.

#### Regularization

Since the inverse problem of electrocardiology is ill-posed, measurement and modeling noise will cause substantial deviations from the true solution. In the EDL-based inverse, this is limited by not minimizing RD (the relative difference between simulated and reconstructed ECGs, see section 2.5), but by minimizing RD+*λ* ∙ REG, where REG is the regularization function. For depolarization, this function is defined as the root mean square of the Laplacian of the depolarization times at the heart surface elements, multiplied by the square root of the surface area of these elements as follows:

$$ \mathrm{REG}=\sqrt{\sum \limits_{i=1}^{N_s}{\left(\Delta {\tau}_i\sqrt{a_i}\right)}^2}, $$

with *τ*_i_ the activation time at node *i*, *a*_i_ the surface area at node *i*, and *N*_s_ the number of nodes at the heart surface. Defined this way, REG is independent of the number of nodes in the mesh, as well as the size of the heart (provided that activation propagation velocity is the same for all heart sizes). For repolarization, REG is defined analogously.

As the L-curve method to find the optimal value for lambda often leads to over-regularization [3], we set beforehand a target value for REG that results in physiologically realistic timing patterns. We found that a REG value of 25 s/m corresponds to realistic activation patterns, while a REG value of 10 s/m corresponds to realistic repolarization patterns. We assume this to be an inherent property of the (healthy) heart. Consequently, we selected a value for the regularization parameter λ_dep_ and λ_rep_ that results in REG ≈ 25 s/m and 10 s/m, respectively.

### Determining robustness

We determined the robustness of the inverse method by changing one of two factors; (i) tissue conductivities or (ii) amount of noise on body surface signals.

(i) In the forward sense (upper part of Fig. 1), the tissue conductivity values were taken from Gabriel et al. [10]. The inverse calculation (lower part of Fig. 1) was either done with the same conductivity values or with other conductivity values provided in the literature [17] (Table 1), performed on the body surface ECG with baseline noise (20 μV Gaussian white noise).

**Table 1:** Conductivity values used to construct different transfer matrices (A), used in the EDL-based inverse, based on common differences in measured values summarized in [8, 17]. Transfer matrix A uses same conductivity values as used in the construction of the simulated body surface potentials

Tissue type	Conductivity [S/m]				
	*A*	*A1*	*A2*	*A3*	*A4*
Thorax	0.2	0.2	0.2	0.2	0.2
Lungs	0.04	0.06	0.06	0.01	0.06
Blood	0.6	0.6	0.6	0.6	0.75
Heart	0.2	0.1	0.4	0.2	0.3

(ii) The forward simulated body surface ECG was by default contaminated with 20 μV Gaussian white noise, which was increased to either 40, 60, or 80 μV Gaussian white noise.

### Data presentation

The root-mean-square error (RMSE) and correlation (COR) between simulated and reconstructed repolarization times were calculated, as well as interquartile ranges (IQR) of absolute differences. In addition, the COR and relative difference (RD) values between the simulated and inverse reconstructed body surface ECGs were determined. The RD was defined as the Frobenius norm of the difference between the simulated and reconstructed signals relative to the Frobenius norm of the simulated data.

## Results

### Regular cases without addition of errors or noise

**Fig. 2:**
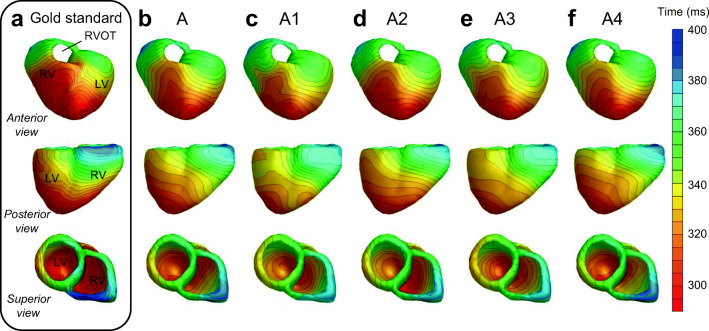
Repolarization patterns for beat in sinus rhythm. **a** is the actual repolarization pattern used to calculate the body surface maps, **b** is the repolarization pattern reconstructed with the inverse procedure. **c**–**f** are the repolarization patterns reconstructed with the inverse procedure when a transfer matrix with different conductivity values is used, given in Table 1. The following anatomical landmarks are given in panel a: right ventricle (RV), left ventricle (LV),and right ventricular outflow tract (RVOT)

**Fig. 3:**
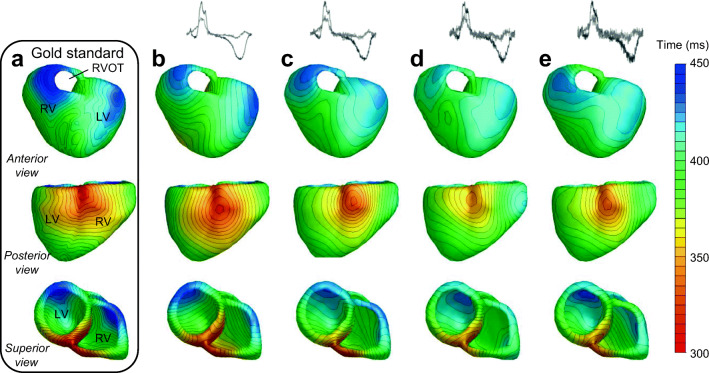
Repolarization patterns for beat 2 (ectopic beat with origin on base of right ventricle). **a** is the actual repolarization pattern used to calculate the body surface maps, **b**–**e** represent the repolarization pattern reconstructed with the inverse procedure with different amplitudes of (Gaussian white) noise added to the body surface potentials (20, 40, 60, and 80 μV for column b, c, d and e, respectively). Above panels b through e, two leads of the body surface ECG are shown with the different amounts of noise added. The following anatomical landmarks are given in panel a: right ventricle (RV), left ventricle (LV), and right ventricular outflow tract (RVOT)

**Fig. 4:**
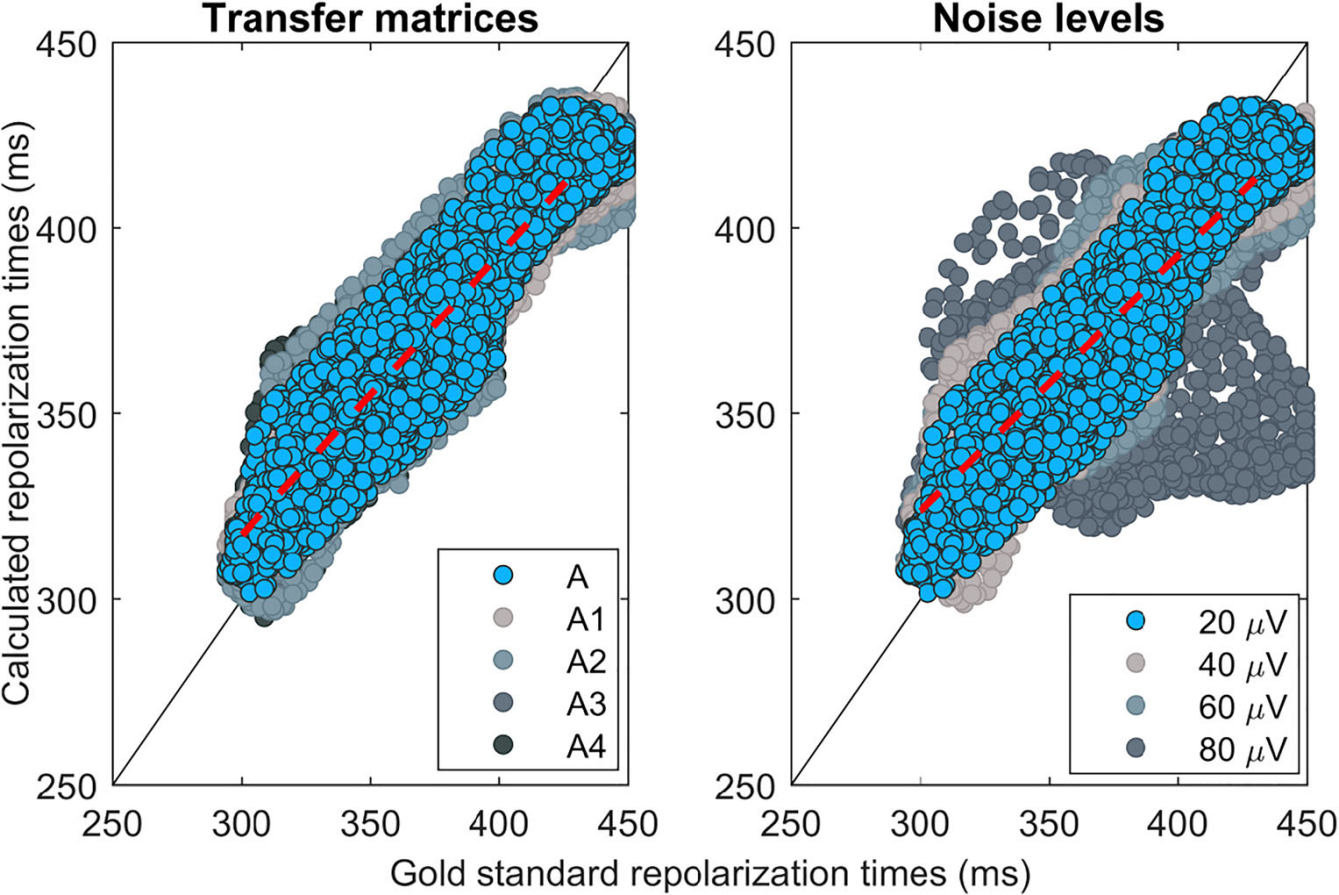
Scatter plot of inverse reconstructed repolarization times matched with the gold standard repolarization times for all nodes. Left shows the results for different tissue conductivities and right the results for different levels of noise added to the body surface ECGs. Note that the series of blue dots (i.e., transfer matrix A and 20 μV of noise) is the same series, therefore displayed in both left and right plot

**Fig. 5:**
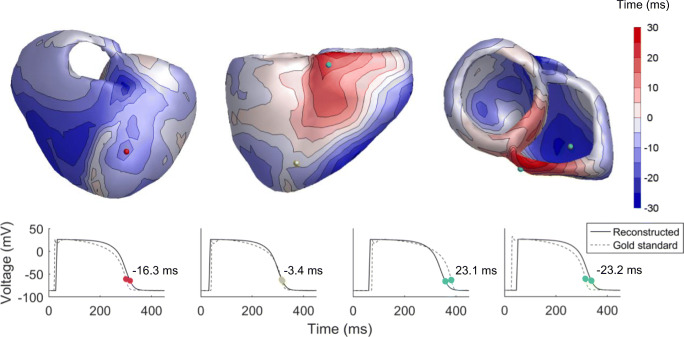
Differences between simulated and reconstructed repolarization times for beat 1, with normal transfer matrix and 20 μV of noise (simulated minus reconstructed). Bottom part shows corresponding simulated (dashed) and reconstructed (solid) TMPs at the locations indicated by the colored spheres, with in each graph the repolarization time difference between the two

The repolarization times at the ventricular surface that resulted from the monodomain model for a sinus beat is shown in Fig. 2 a, while the inverse estimated repolarization pattern in shown in Fig. 2 b. Figure 3 and Supplementary Fig. 1 show the results for ectopic ventricular activations beat 2 and beat 3 in the same format, respectively. Without addition of errors and noise, all three beats show an inversely reconstructed repolarization pattern that is very similar to the original pattern, with only small differences. There is a good correlation between the repolarization patterns in all beats, showing early and late repolarization in the same regions in the heart (Table 2). Similarly, the RMSE is low (12.0, 12.2, and 15.3 ms for beats 1, 2, and 3, respectively). A difference between the reconstructed pattern and gold standard is that the range of the reconstructed repolarization times is smaller. In the gold standard, the range in repolarization times (min-max) is 101, 127, and 146 ms for beats 1, 2, and 3, respectively, while it is 93, 109, and 101 ms in the reconstructed patterns. The RMSE was similar for endocardial and epicardial nodes in beats 1 and 2, and higher in beat 3 (12.3 vs. 11.5 ms in beat 1, 12.8 vs. 11.6 ms in beat 2, and 17.4 vs. 12.6 ms in beat 3 for endocardial and epicardial nodes, respectively). Figure 4 shows the association between original and inverse reconstructed repolarization times in blue, and for the inverse reconstructions with added inaccuracies in gray. It shows a good overall correlation, with *R* = 0.87 (*p* < 0.01). Table 2 summarizes the correlation data for all reconstructions. The differences between gold standard and reconstructed repolarization pattern for beat 1 (Fig. 2 column a–column b) can be seen in Fig. 5. The bottom part of this figure shows the simulated TMP (dashed) and reconstructed TMP (solid) on four different locations on the heart.

**Table 2:** Comparison of repolarization mapping accuracy with the use of different transfer matrices

Repolarization pattern	Used transfer matrix	λ_rep_	RMSE (ms)	IQR (ms)	COR rep pattern	COR ECG	RD ECG
Beat 1 (sinus)	A	8e-7	12.0	4.3–14.7	0.89	0.99	0.13
	A1	1.5e-6	14.5	5.5–18.4	0.88	0.99	0.14
	A2	1.5e-6	11.8	4.2–14.4	0.90	0.99	0.13
	A3	1.5e-6	13.2	5.1–17.2	0.88	0.99	0.14
	A4	7e-7	13.3	5.2–16.3	0.87	0.99	0.13
Beat 2 (ectopic base RV)	A	1e-5	12.2	4.0–15.0	0.93	0.99	0.14
	A1	7e-6	12.4	3.8–14.9	0.93	0.99	0.13
	A2	1e-5	13.0	3.8–15.7	0.93	0.99	0.15
	A3	9e-6	11.8	4.0–14.5	0.94	0.99	0.14
	A4	1e-5	12.8	3.8–15.3	0.93	0.99	0.15
Beat 3 (ectopic left side septum)	A	2e-6	15.3	4.2–18.0	0.92	0.99	0.15
	A1	4e-6	15.0	4.4–16.8	0.94	0.99	0.15
	A2	2e-6	17.3	4.5–19.2	0.87	0.99	0.16
	A3	2e-6	14.3	4.0–16.5	0.95	0.99	0.14
	A4	2e-6	17.2	4.7–19.3	0.87	0.99	0.15

### Robustness: Tissue conductivities

Figure 2 b–f show the reconstructed patterns following the inverse procedure with the five different conductivity settings from Table 1 during a sinus beat. Application of the five different transfer matrices results in similar patterns, with early repolarization in the apex and late repolarization in the RV lateral base. The main difference between the results for the transfer matrices is the smoothness of the patterns (e.g., least smooth pattern when using transfer matrix A1, panel c). The correlations between inverse reconstructed patterns and gold standard was similar in all cases, ranging from 0.87 to 0.90. The RMSE was around 12 ms, and increased to a maximum of 14.5 ms when using transfer matrix A1 (Table 2). This is similar for the two ectopic beats (Supplementary Fig. 1 and 2), with good correlation and low RMSE. Only A2 and A4 in beat 3 (panel d and f in Supplementary Fig. 1) have a correlation that is slightly below 0.90.

### Robustness: Noisy body surface ECGs

Figure 3 b–e show the reconstructed patterns following the inverse procedure with 20, 40, 60, or 80 μV of noise added to the body surface ECG for beat 2. There is a slight increase in pattern error with increasing noise, but correlations remain higher than 0.85 with a maximum RMSE of 18 ms (Table 3). Only the reconstruction with 80 μV of noise for beat 3 gives a repolarization pattern showing a very low correlation with the gold standard pattern and a high RMSE. Supplementary Table 1 shows a comparison of the results in relation to robustness of the activation pattern. It shows that reconstruction of activation and repolarization has a similar accuracy in estimating the gold standard pattern.

**Table 3:** Comparison of repolarization mapping accuracy in the presence of different levels of noise on the body surface potentials

Repolarization pattern	Noise added to body surface ECG	λ_rep_	RMSE (ms)	IQR (ms)	COR rep pattern	COR ECG	RD ECG
Beat 1 (sinus)	20 μV	8e-7	12.0	4.4–14.7	0.89	0.99	0.13
	40 μV	1e-6	13.4	5.1–16.4	0.86	0.98	0.21
	60 μV	1.2e-6	16.0	8.3–19.0	0.81	0.97	0.30
	80 μV	4e-6	18.9	9.1–23.9	0.79	0.93	0.41
Beat 2 (ectopic base RV)	20 μV	1e-5	12.2	4.0–15.0	0.93	0.99	0.14
	40 μV	2e-6	12.9	4.2–15.5	0.92	0.99	0.18
	60 μV	2e-6	17.9	5.9–22.2	0.85	0.97	0.31
	80 μV	2e-6	15.4	5.0–18.7	0.88	0.97	0.31
Beat 3 (ectopic left side septum)	20 μV	2e-6	15.3	4.3–18.0	0.92	0.99	0.15
	40 μV	2e-6	18.0	5.9–20.7	0.89	0.99	0.20
	60 μV	2e-6	17.7	5.0–21.4	0.88	0.97	0.28
	80 μV	4e-6	45.6	13.8–49.6	0.05	0.94	0.36

## Discussion

In the present in silico study, we investigated the performance of the inverse reconstruction of repolarization times using the EDL-based method to reconstruct epicardial and endocardial repolarization times. The results showed that the EDL model correctly reconstructs the repolarization patterns (correlation range 0.85–0.95). The adaptation of tissue conductivities in the transfer matrix and addition of noise to the body surface ECG demonstrated that the inverse repolarization reconstructions were robust, showing good correlations with the gold standard repolarization times. The accuracy and robustness of reconstructed repolarization patterns were similar to the accuracy and robustness of the activation patterns under the same conditions.

### Equivalent dipole layer inverse

This is the first study showing that the adaptation of the EDL method to include repolarization (i.e., from uniform dipole layer to non-uniform dipole layer with strength proportional to the local TMPs) leads to accurate results when inversely reconstructing the cardiac repolarization times. This advance is potentially clinically relevant, because repolarization disorders underlie life-threatening cardiac arrhythmias [5] and non-invasive detection of local repolarization changes is therefore important for diagnosis risk stratification and the evaluation of therapy.

The shape of the TMP, i.e., the slope of decrease in amplitude during repolarization, was determined by the dominant T-wave on the body surface ECG. This allows individualization of the TMP shape, thereby increasing the probability of a correct solution. This method lacks the ability to model the phase 1 notch in TMP caused by the transient outward current of potassium. Given that this notch occurs immediately after depolarization, it may influence both QRS complex and T-wave on body surface ECG. In this study, the phase 1 notch was included in the simulated TMPs used for forward calculation of the ECGs (as can be seen in the TMPs in Fig. 5). The good correlations and low RMSE of the inversely reconstructed repolarization times show that this difference in TMP template does not largely influence the accuracy of the EDL method.

The current regularization method used a regularization parameter λ_rep_ in such a way that the REG value will be around 10 s/m. This results in patterns that are smooth enough to be realistic without having small areas of extreme values for repolarization times. However, for diseased hearts, it may be necessary to allow larger values for regularization in order to detect subtle repolarization heterogeneities. For example, in hearts with sharp repolarization gradients, smoothing these gradients by regularization is not a desired effect. Previous knowledge of the patient’s status could help finding a physiologically valid repolarization pattern, although this is susceptible to bias.

### Previous studies

Previous inverse validation studies using the EDL model have shown good results in in silico, ex vivo, and in vivo validation for activation, with activation timing errors between 5 and 20 ms [15, 23, 24] and localization errors of the origin of ectopic beats between 2 and 25 mm [15, 24]. To our best knowledge, ours is the first study that quantifies the EDL method accuracy for repolarization times. In a study by van Dam et al., the EDL model was used to reconstruct repolarization sequences without comparison with a gold standard [6]. In that study, repolarization patterns were consistent with prior physiological knowledge. In the present study, we were able to directly compare EDL inverse reconstructed repolarization times with the actual repolarization times on all nodes in the heart. We found error and correlation values that are similar to values reported for inverse activation reconstruction.

The accuracy of potential-based inverse reconstruction of repolarization has been previously investigated. Cluitmans et al. reported a correlation between measured and reconstructed repolarization times of 0.73, measured in four dog hearts at 103 electrode positions [4]. They described that the correlation improved by determining the repolarization times with a spatiotemporal approach instead of a temporal-only approach. This step is not necessary in the EDL-based inverse method since it directly calculates activation and repolarization times. There are also studies describing repolarization reconstruction accuracy in humans. In a study by Zhang et al., epicardial potentials were measured in patients undergoing open-heart surgery. They then used a similar approach as we have applied by calculating the ECG input for the inverse calculation based on the measured epicardial potentials [39]. They describe that 78% of the 240 compared epicardial electrograms had a difference between reconstructed and measured activation recovery intervals of less than 10 ms with the epicardial potential method. A study by Graham et al. directly compares reconstructed epicardial repolarization patterns with CARTO electroanatomical maps in patients with structural heart disease, and found correlation of repolarization sequences of 0.55 and RMSE of 51 ms [13]. Although it is difficult to directly compare these studies with the current study, our study shows results that are equivalent or even superior to the results from the other studies. This can be explained by the differences between the inverse method (potential-based versus EDL) and by the fact that the current study is an in silico study. In the in silico study, the conditions are better controlled than in the in vivo studies. However, we applied various forms of error to simulate in vivo conditions.

### Robustness of the EDL-based inverse method

Tissue conductivity is a large determinant factor for the relation between electrical activity on the heart and electrical activity (potentials) at the body surface, although exact individual tissue conductivity values are not certain [8] or are influenced by pathological conditions [28, 31]. In the current study, we have investigated the influence of tissue conductivity on the inverse procedure by applying various conductivity values. These values differed from the conductivity values used in the FEM-forward calculation of the simulated body surface ECGs. This approach is equivalent to assuming a conductivity value in patient tissue that deviates from the actual conductivity of the tissue. Our results show that the effects of these discrepancies in tissue conductivity have a small effect on the accuracy of the inverse reconstruction. This corresponds to results from Bear et al. for inverse estimation of epicardial activation potentials [2], showing that inclusion of inhomogeneous torso electrical properties (i.e., tissues with different conductivities) did not improve accuracy of the inverse calculation of activation as compared with inverse calculation ignoring these inhomogeneities.

The introduction of noise on the body surface ECGs could cause problems for the inverse method due to the ill-posedness of the problem [1, 14]. The noise levels applied in this study were related to signal-to-noise ratios (SNR) ranging from 20 dB for low noise level up to 6 dB for high noise level. Average SNR found in human body surface mapping are around 13 dB [19]. These high levels of noise severely influence the body surface ECG, thereby even masking the T-wave (Fig. 3, top panels). The SNR for beat 3 at 80 μV being lower than the SNR for beats 1 and 2 at high noise level (average SNR of 6 dB for beat 3 versus 9 dB for both beats 1 and 2) may therefore be an explanation for the bad performance of beat 3 at 80 μV of noise. The results of the present study show that the addition of Gaussian white noise of up to 80 μV did not lead to a significant inaccuracy of the reconstructed repolarization pattern.

### Limitations

In the present work, we aimed to determine the robustness of the inverse solution by using different tissue conductivities for forward and inverse computations and by adding more noise to the simulated body surface ECGs. Other factors, e.g., geometry and electrode positions, remained constant for both forward and inverse computations, thereby possibly leading to certain model-to-model bias. However, different discretization between forward and inverse calculations (FEM versus BEM), parameterization of TMP for inverse calculation without inclusion of phase 1 dip and agreement of the results with the previous in vivo EDL validation study on depolarization gives credibility to the results of the present study.

Mechanical contraction of the heart has been shown to alter the T-wave on the body surface ECG [18, 35, 37]. In the present study, changes in geometry due to cardiac contraction, blood flow, and respiration were not taken into account (both in forward calculation of simulated ECGs as in inverse calculation of repolarization times). In a human or animal study using inverse methods, this motion will be present, especially during repolarization, and will therefore influence the projection of repolarization electrical activity on the body surface [20].

### Clinical application

The EDL-based inverse can be used to determine activation and repolarization patterns from patients at risk for cardiac arrhythmias (e.g., due to (family history of) arrhythmogenic syndromes). Interpretation of the individual patterns can provide (additional) topical information into treatment strategies, such as ablation approaches and ICD implantation. Determination of size and location of electrical heterogeneities may improve success rates of ablation procedures while simultaneously lowering costs by reducing procedure time. In addition, the reconstruction of both the endocardial and the epicardial distribution of repolarization by the EDL-based method can reduce procedure time even more by being able to start the procedure with the correct (i.e., epicardial or endocardial) initial approach.

## Conclusion

This in silico validation study shows that the non-invasive ECG inverse solution using the EDL model has good accuracy of repolarization pattern reconstruction, even in the presence of model disturbances and noise. Together with the (previously validated) depolarization patterns, better visualization of the complete electrical activity of the individual patient can be achieved. This will help improve understanding of cardiac arrhythmias and may guide clinical decision-making.

## Electronic supplementary material

ESM 1 (DOCX 924 kb)
