# Supplementary material for: In silico validation of electrocardiographic imaging to reconstruct the endocardial and epicardial repolarization pattern using the equivalent dipole layer source model
Source: Med Biol Eng Comput. 2020 May 31;58(8):1739–49. doi: 10.1007/s11517-020-02203-y (PMC7340677; doi:10.1007/s11517-020-02203-y)
Supplement: Supplementary file 1 — (DOCX 924 kb) [file 11517_2020_2203_MOESM1_ESM.docx]

## **Supplementary material**

**In silico validation of electrocardiographic imaging to reconstruct the endocardial and epicardial repolarization pattern using the equivalent dipole layer source model**

Jeanne van der Waal^1^, Veronique Meijborg^1^, Ruben Coronel^1*^, and Thom Oostendorp^2*^

J. van der Waal (correspondence email: j.g.vanderwaal@amsterdamumc.nl), V. Meijborg and R. Coronel are with the Department of Clinical and Experimental Cardiology, Amsterdam University Medical Centers, Amsterdam, the Netherlands.

T. Oostendorp is with The Donders Institute for Brain, Cognition and Behaviour, Radboud University Medical Centre, Nijmegen, The Netherlands.

This research is supported by a grant from the Leducq Foundation

^a^Ruben Coronel and Thom Oostendorp contributed equally to this work.

*Keywords*—Inverse problem, Electrocardiographic Imaging, Equivalent Dipole Layer, Repolarization, Cardiac Arrhythmias.

^1^ Department of Clinical and Experimental Cardiology, Amsterdam University Medical Centers, Meibergdreef 9, 1105 AZ Amsterdam, the Netherlands

^2^ Donders Institute for Brain, Cognition and Behaviour, Radboud University Medical Centre, Kapittelweg 29, 6525 EN Nijmegen, the Netherlands

^*^ Ruben Coronel and Thom Oostendorp contributed equally to this work

Corresponding author: Jeanne van der Waal, j.g.vanderwaal@amsterdamumc.nl , +3120 5663269


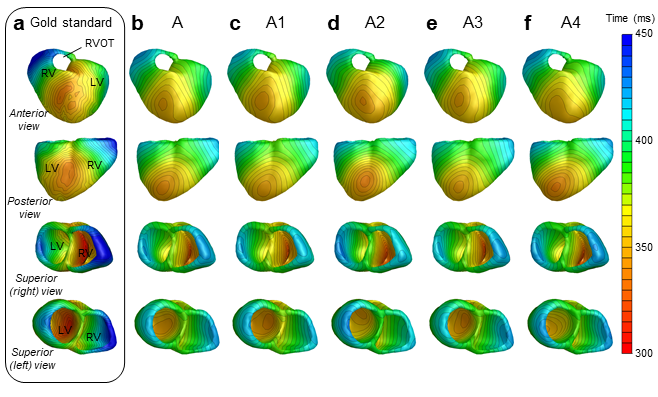


**Supplementary figure 1** Repolarization patterns for beat 3 (ectopic rhythm – origin on left side of septum). Column A is the actual repolarization pattern used to calculate the body surface maps with, column B is the repolarization pattern calculated with the inverse procedure. Columns C – F are the repolarization patterns calculated with the inverse procedure when a transfer matrix with different conductivity values is used, given in Table 1


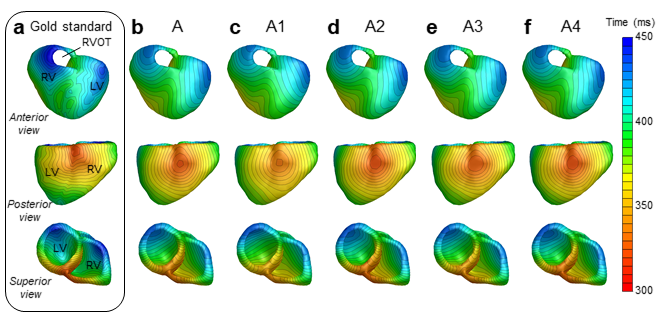


**Supplementary figure 2** Repolarization patterns for beat 2 (ectopic rhythm – origin on base RV near septum). Column A is the actual repolarization pattern used to calculate the body surface maps with, column B is the repolarization pattern calculated with the inverse procedure. Columns C – F are the repolarization patterns calculated with the inverse procedure when a transfer matrix with different conductivity values is used, given in Table 1


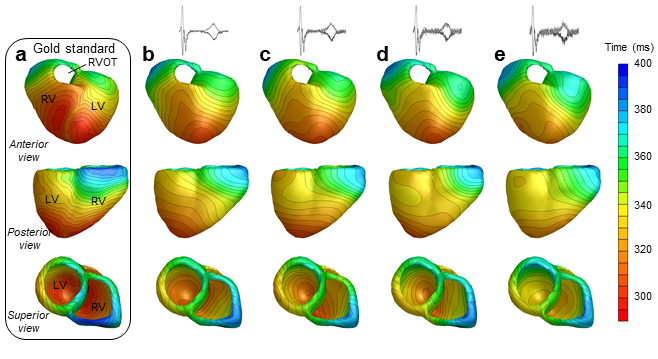


**Supplementary figure 3** Repolarization patterns for beat 1 (sinus). Column A is the actual repolarization pattern used to calculate the body surface maps with, columns B through E represent the repolarization pattern calculated with the inverse procedure with different levels/amplitudes of (Gaussian white) noise added to the body surface potentials (20, 40, 60 and 80 μV for column B, C, D and E respectively)


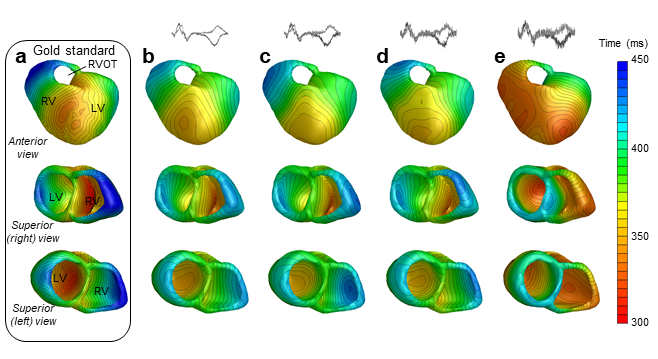


**Supplementary figure 4** Repolarization patterns for beat 3 (ectopic beat with origin on left side of septum). Column A is the actual repolarization pattern used to calculate the body surface maps with, columns B through E represent the repolarization pattern calculated with the inverse procedure with different levels/amplitudes of (Gaussian white) noise added to the body surface potentials (20, 40, 60 and 80 μV for column B, C, D and E respectively)

**Supplementary table 1** Comparison of accuracy in the presence of different levels of noise on the body surface potentials for activation and repolarization.

|  | Noise added to body surface ECG | RMSE **act** [ms] | Cor **act** pattern | RMSE **rep** [ms] | Cor **rep** pattern |
| --- | --- | --- | --- | --- | --- |
| Beat 1 (sinus) | 20 µV | 17.2 | 0.73 | 12.0 | 0.89 |
|  | 40 µV | 25.1 | 0.63 | 13.4 | 0.86 |
|  | 60 µV | 24.7 | 0.64 | 16.0 | 0.81 |
|  | 80 µV | 17.9 | 0.73 | 18.9 | 0.79 |
| Beat 2 (Ectopic base RV) | 20 μV | 17.1 | 0.88 | 12.2 | 0.93 |
|  | 40 μV | 14.7 | 0.91 | 12.9 | 0.92 |
|  | 60 μV | 17.5 | 0.90 | 17.9 | 0.85 |
|  | 80 μV | 14.7 | 0.91 | 15.4 | 0.88 |
| Beat 3 (Ectopic left side septum) | 20 μV | 17.4 | 0.88 | 15.3 | 0.92 |
|  | 40 μV | 17.3 | 0.88 | 18.0 | 0.89 |
|  | 60 μV | 18.1 | 0.89 | 17.7 | 0.88 |
|  | 80 μV | 43.1 | 0.45 | 45.6 | 0.05 |
